# Evaluation of alternate outreach models for cataract services in rural Nepal

**DOI:** 10.1186/1471-2415-10-9

**Published:** 2010-03-25

**Authors:** Ram P Kandel, Sowmya R Rajashekaran, Maria Gautam, Ken L Bassett

**Affiliations:** 1 Seva Foundation/Seva Canada Society, Nepal Eye Hospital Complex, Tripureswor, Kathmandu Nepal; 2 Research Department, Lumbini Eye Institute/Shree Rana Ambika Shah Eye Hospital, Bhairahawa Nepal; 3 Ophthalmologist, Bharatpur Eye Hospital, Chitwan Nepal; 4 Department of Ophthalmology, British Columbia Centre for Epidemiologic & International Ophthalmology, 1081 Burrard Street, Vancouver BC V6Z Y6 Canada

## Abstract

**Background:**

Bharatpur Eye Hospital in Chitwan District, a primarily agrarian setting in south-central Nepal, reduced the number of diagnostic screening and treatment (DST) camps by one half (151 to 75) in an attempt to increase both the efficiency of its outreach program and the number of people that go directly to the hospital for service. The Hospital evaluated the two program models in terms of program costs, cataract surgical utilization, hospital direct payment and patient equity.

**Methods:**

The study is a prospective, before and after, study of the impact of an alternate outreach model on cataract service utilization patterns and cost per outreach camp and cost per cataract surgery at Bharatpur Eye Hospital, comparing the service years July 2006 to June 2007, with July 2007 to June 2008. Study findings were based on routinely gathered hospital and outreach administrative data.

**Results:**

The total cost of the DST camps decreased by approximately US$2000. The cost per camp increased from US$52 to $78 and the cost per cataract surgery decreased from US$ 3.80 to $3.20. The number of patients who went directly to the hospital, and paid for cataract surgery, increased from 432 (17%) to 623 (25%). The total number of cataract surgical procedures at Bharatpur Eye Hospital remained very similar between the two service years (2501 and 2449, respectively). The presenting visual acuity and sex of the two cataract surgical populations were very similar (favouring women, 53 and 55% in the two years, respectively). A shift toward younger men and women occurred with a 245 (64%) increase in people age 50-59 years, and shift away from people age 70 years and older with a 236 (22%) reduction. The age and sex distribution of the direct paying patients were very similar in the two years.

**Conclusion:**

The new, more concentrated, more rural DST model of service delivery reduced overall outreach program costs, cost per cataract surgery transported, while increasing direct payments to the hospital, with a significant decrease in the number of people age 70 and older in the first year.

## Background

Hospital-based eye care programmes increasingly need to connect more actively and equitably with communities, rather than passively wait for patients to come to them. In Nepal, this is accomplished through a combination of village-level primary eye care centres with outreach services such as school screening programs, and diagnostic-screening and treatment (DST) camps.

Most DST camps in Nepal follow what is commonly know as the Aravind model [1]. A team with or without an ophthalmologist, but always a skilled ophthalmic assistant, makes a planned, advertised visit to a community and provides basic ocular care, identifies patients who need cataract surgery, and offers them transport back to the base hospital for the operation. This model is seen to have several advantages over surgical eye camps because it minimizes the time the ophthalmologist is away from and maintains the best quality surgery at the base hospital. While eye care programs take various approaches to these DST outreach strategies, they almost never critically examine alternate program options.

Bharatpur Eye Hospital, in south-central Nepal, employs two full time ophthalmologists and conducts approximately 2500 cataract surgical procedures per year under the supervision of the Lumbini Eye Institution. It provides DST camps throughout the Chitwan and Nawalparasi Districts. The hospital lies at the boarder of the two Districts, separated by a major river (Narayan). Approximately 40% of the population lives below the poverty line.

Three years ago, Bharatpur Eye Hospital began to more systematically plan and evaluate its community outreach program. The program, while utilizing a well established DST camp model [1], conducted the DSTs in places more out of historical convenience than based on demographic planning. DSTs had, in fact, been added over time in virtually any community requesting one, if the local community provided volunteer support for advertising and logistics. DSTs, which provide free consultation and access to cataract surgery, also occurred within a few kilometres of the hospital, often attended by wealthier people.

The Hospital planned fewer, more strategic DST camps to increase the efficiency of its outreach program. The hospital reduced the number of DSTs in or near Bharatpur partly because it was concerned with equity. They wanted people from higher socio-economic classes, living near to the hospital, to pay for surgery, if they could. The hospital also sought to increase the number of 'direct' paying people to improve its financial sustainability. People who come directly to the hospital usually pay the cost of cataract surgery, unlike people referred from DST camps, where cataract surgical costs are free. At no time did the Hospital actively encourage direct paying patients through public advertisements or seek referrals from the primary health care system.

Along with DST program planning, the Hospital recognized the need to build their capacity to conduct ongoing assessment of its community eye care system and the services it provided, including cost. The Hospital therefore initiated this evaluation of the two DST program models in terms of program costs, cataract surgical utilization, hospital direct payment and patient equity.

## Methods

This is a prospective, before-and-after, evaluation of two community program models with different number, duration, and location of DST camps.

The Hospital administrative data included patient demographics (age, sex, address) payment, clinical diagnosis, hospital procedures, as well as presenting and discharge visual acuity. The administrative data was entered in and reported by the Aravind Eye Care System softwear, modified for a secondary level eye care facility.

The Hospital altered its administrative system to allow for attribution of hospital staff time, equipment, supplies, and vehicles specifically to the community outreach program. This meant calculating a portion of annual salaries and expenses (such as vehicle license and maintenance) and direct recording of supplies used and travel costs incurred.

The Hospital did not routinely calculate the specific expenditures and income from cataract surgery, or any other program. Instead, it calculated its total operating costs, including building maintenance, medical equipment, salaries and supplies. The Hospital then calculated its income-generating, versus free, cataract services as a percentage. The Hospital set targets for direct payment for cataract surgical services as part of its goal of financial self-sustainability.

The Hospital also did not have an established system to determine the expenses and income from individual departments, such as the optical shop or drug dispensary. Instead, one system gathered and totaled all patient fees paid and government subsidies received, while a separate system gathered and totaled all expenses, such as purchase of supplies. The department specific contributions to total Hospital operating costs included herein, therefore, are general estimates, not actual expenditures.

The Hospital established, *a priori*, that indicators of success included a decrease in outreach program costs, stable or increased number of overall cataract operations, with less than a 5% change in the proportion of women, elderly or poor patients. The Hospital did not set a specific goal for an increase in 'direct' (sought hospital services on their own) paying patients, although their long-term target was to have 33% of patients pay for cataract surgery 'directly'.

The primary efficacy measure was the outreach program cost per cataract surgery performed at Bharatpur Eye Hospital. The program evaluation sought data to establish baseline and numerical trends over time, beginning with this two year period. Statistical comparisons therefore were not planned for data from these two years.

### Applicability of cost estimates

1. Costs computed were for identifying patients through DST camps and performing free surgery at the Bharatpur Eye Hospital.

2. Costs computed were applicable only to DST camps conducted within 60 kms radius of the Bharatpur Eye Hospital.

3. A cataract patient, for the purposes of this study, was a patient with operable cataract who accepted immediate surgery at Bharatpur Eye Hospital.

4. Per patient costs were computed based on the total number of cataract patients as defined in No. 3 above;

5. At some DSTs, registration fees were collected by the local voluntary agency conducting the camp. The registration fees were not included in the analysis.

6. The calculation of DST camp costs included all dimensions of providing primary eye care diagnosis and treatment. Other surgical procedures that add additional costs and other eye care treatment that confers additional benefit were not considered herein.

### DST camps and cataract surgery at Bharatpur Eye Hospital

1. A schedule is developed based on various demographic and program factors.

2. Clubs, organizations, and administrators in the local community are contacted and asked to organize a venue, arrange for volunteers, and provide advertisement.

3. Publicity includes pamphlets, radio announcements, as well as verbal support from village leaders and teachers. The pamphlets and messages clearly state whether or not there is a need to pay for services.

4. During the DST camps, all people who register are tested for visual acuity. Minor eye conditions are treated, glasses are sold, and patients with operable cataract are advised to have surgery.

5. Cataract blind people (central lens opacity and visual acuity of 6/60 or less) are offered counselling by female community health volunteers.

6. Cataract patients who accept immediate surgery receive free transportation to the hospital with hospital vehicles and free cataract surgery usually the next day.

7. The entire cost of surgery is free for the DST camp patients including hospital stay, except for postoperative medicines, dark glasses, food, and transport home from the hospital (total $US 3).

### Change in DST camps

From July 2006 to June 07, Bharatpur Eye Hospital conducted 151 DST camps spread throughout Chitwan and Nawalparasi Districts. The average distance to hospital was 30 kilometers (range 3 to 60 kilometers) with 11 DST camps held in the urban area (Bharatpur municipality). In 30 sites, 2 DST camps were held in the same location within a 6 month period.

From July 07 to June 08, the Hospital reduced the number of DST camps to 75, 70 of which were held in rural areas. The 5 DSTs that occurred in Bharatpur municipality were conducted in more remote areas not served in the previous year.

The average distance from the rural DSTs to the base hospital remained the same between the two years. The primary difference was that fewer DSTs were conducted per region with increased publicity to more villages surrounding the DST site. Additional publicity included more pamphlets and poster as well as a full day of advertising from a roving vehicle the day prior to the DST.

In 2007/08, while each DST still lasted one day, the new strategy involved, at times, conducting 2 to 4 DSTs on the same trip. In 2006/07, the team returned to Bharatpur between each DST. In 2007/08 the team also returned more than once to only 8 sites, as opposed to 30 sites the year before.

## Results

From 2006/07 to 2007/08 there was a decrease in the number of person-days that staff were away from the hospital, from 342 to 300 person-days (Table 1). There was an increase in the number of staff per camp from 2 to 4. There was also a 27% increase in salary and per diem in 2007/08. Overall, approximately US$2000 were saved by providing fewer DST camps in 2007/08.

**Table 1:** Costs of Diagnostic Screening and Treatment Camps 2006-07 and 2007-08

Year	2006/07	2007/08
Per diem*	107,664	93,775
Transportation expenses	231685	125852
Salary for Ophthalmic Assistants and Nurses**	103,445 (for 171 days)	122,642 (for 75 days)
Publicity	9,547	17,458
Other expenses	8,820	7,738
Transportation including vehicle depreciation, repair and insurance***	90142	41792
**Total**	**551303 (US$7875)**	**409257 (US$5,846)**
DSTs	151	75
Cost per DST	3651 (US$52.15)	5456 (US$77.95)
Total cataract patients transported for surgery	2069	1826
Average cataract surgical patients transported per DST	14	24
**Cost per cataract patient transported**	**3.80 (US$)**	**3.20 (US$)**

* Per diem is a daily wage supplement paid in addition to regular salary while away from the hospital

** In 2007/08 the salary for hospital staff increased by 27% and the number of people per DST increased from 2 to 4. As a result, DST camps in 06/07 had 342 person-days, while DSTs in 07/08 had 300 person-days.

*** In each year the total cost of maintenance, insurance and fuel was divided by the number of days the vehicle was with the DST i.e for 2006/07, 151 DST days = 151/365 (41%) of annual cost; for 2007/08, 75 DST days = 75/365 (20%) of total annual cost.

*** Exchange rate US$ 1 = Nrs.70

With 151 DST camps in 2006/07 and 75 in 2007/08 the cost per camp increased from US$52 to $78, respectively, and the cost per patient transported for cataract surgery decreased from US$3.80 to US$3.20 (Table 1).

The total number of cataract surgical procedures at Bharatpur Eye Hospital remained virtually the same, decreasing by 52 patients [2%] in 2007/08 (Table 2). The age and sex of the two cataract surgical populations showed an overall numerical increase (9%) in the number of people in the age 50-59 age group for both men and women, mainly at the expense of the over 70 age group, which decreased 9%. Among older people, there was a 236 person (22%) reduction in the number served, and among younger people there was a 245 person increase (64%). The proportion of men and women receiving services remained similar, favouring women 53% and 55%, respectively.

**Table 2:** Cataract surgery by age and sex for 2006-07 and 2007-08

	2006/2007			2007/2008		
Age (Years)	Men	Women	Total N(%)	Men	Women	Total N(%)
< 50	71	98	**169 (7)**	61	106	**167 (7)**
50-59	164	220	**384 (15)**	290	339	**629 (26)**
60-69	435	463	**898 (36)**	361	478	**839 (34)**
70+	503	547	**1050 (42)**	378	436	**814 (33)**
**Total**	**1173 (47)**	**1328 (53)**	**2501 (100)**	**1090 (45)**	**1359 (55)**	**2449 (100)**

The presenting visual acuity of the two cataract surgical populations were similar except for 10% more women presenting with a visual acuity less than 6/60 and 11% with visual acuity 6/18 or better (Table 3). Both shifts increased the sex ratio favour women.

**Table 3:** Cataract surgery by sex and presenting visual acuity for 2006-07 and 2007-08

2006/2007				207/2008		
Visual acuity	Men	Women	Total N(%)	Men	Women	Total N(%)
Worse than 6/60	51	62	**113 (5)**	73	135	**208 (8)**
6/60-6/24	890	1046	**1936 (77)**	792	888	**1680 (69)**
Better than or equal to 6/18	232	220	**452 (18)**	225	336	**561 (23)**
**Total**	**1173 (47)**	**1328 (53)**	**2501 (100)**	**1090 (44)**	**1359 (56)**	**2449 (100)**

Table 4 provides details of patients who came directly to the hospital in the two years, showing an increase from 432 (17%) to 623 (25%). The age and sex distribution totals were very similar. The only recognizable difference was a 16% increase in the number of women under age 50 (however, the total number of women in this category was only 3% of the population) and a 7% increase in women over age 70.

**Table 4:** Direct payment patients by age and sex for 2006-07 and 2007-08

2006/2007				2007/2008		
Age (years)	Men	Women	Total N(%)	Men	Women	Total N(%)
Less than 50	6	8	**14 (3)**	9	24	**33 (5)**
50-59	61	81	**142 (33)**	82	112	**194 (31)**
60-69	90	116	**206 (48)**	126	153	**279 (45)**
70+	32	38	**70 (16)**	57	60	**117 (19)**
**Total**	**189 (44)**	**243 (56)**	**432 (100)**	**274 (44)**	**349 (56)**	**623 (100)**
**% total surgery**			**432/2501 (17)**			**623/2449 (25)**

## Discussion

Comparing 2007/08 with 2006/07, the reduction in the number of DSTs by 50% had essentially no effect on the total number of cataract surgical procedures. With an estimated US$2000 savings in 2007/08, the cost per cataract surgery performed decreased from US$ 3.80 to $3.20.

Others have examined the cost of non-hospital eye-care services including primary eye care centres or outreach programs. One example comes from a meeting in East Africa where participants from 4 programmes worked together to determine key indicators of the cost of outreach cataract services [2]. Lewallen et al estimated approximately a ten fold higher cost per patient in the East African context US$ 43, with a range from US$ 33 to US$ 56. The lower cost per patient in Nepal is in part due to the lower salary and transportation costs and due to the increased average number of cataract patients transported to hospital per DST camp in Nepal (24) compared with East Africa (14). In both the African and Nepali context, these estimates include providing medical consultation and treatment (not just screening) and basic refractive services.

Aravind Eye Care System found similar costs associated with conducting DST camps but higher transportation costs. They estimated the total cost of case finding of a cataract patient, which included the cost of providing non-cataract related diagnosis treatment, of $US 2.64 and transporting them to and from hospital of $US 3.00 [Personal communication, Aravind Eye Care System. Unpublished, administrative data summary available on request. Accessed November 2008]. Sadguru Netra Chikitsalaya in Chitrakook, northern India estimated the cost of cataract case finding and transportation as US$18.30 [Personal communication, Sadguru Netra Chikitsalaya. Unpublished, administrative data summary available on request. Accessed November, 2008]. The higher cost estimate is seen as due to higher transportation costs and staff salaries.

The reduction in DST camps near to Bharatpur Eye Hospital is associated with an increase in the number of direct paying patients from 17% to 25% of the total number of cataract surgical patients per year. This 8% increase in direct paying cataract surgical patients moved the hospital almost twice that amount (15%) toward service - income self-sufficiency, from 70 to 85%.

The Hospital makes a crude estimate of its income generating self sufficiency. It totals its income for 2007/08, approximately US$ 100,000, which includes subsidies from government and non-governmental organizations, drug and glasses sales, and patient fees. Government subsidies for cataract surgery for people too poor to pay accounted for approximately US$ 27,000. The profit from the sale of eye glasses adds approximately US$ 20,000. The fees derived from people who come directly to the hospital and pay for cataract surgery, the variable considered herein, accounts for about US$ 23,000.

The substantial increase in hospital service-income self-sufficiency is because direct patients pay the 'cost of cataract surgery' 2500 Nepali Rupees (US$35). This payment is in excess of the actual cost to the hospital for medicine, surgical supplies, staff salary and allowances, thereby subsidising patients too poor to pay. Although never really calculated exactly, the hospital sees 70% 'free', 30% 'paying' as necessary for income generating self-sufficiency and 66%/33% as ideal.

The total cost of cataract surgery to patients was more than medical and hospital costs. Additional costs include transportation (likely with a companion) food, and post-operative medicine, estimated as Nepali Rupees 1000 (US$12), for a total of US$47, which is about 30 days of agriculture labour. Willingness to pay the equivalent of one month's labour for cataract surgery is similar to earlier estimates in southern India [1] and more recently in east Africa [3] and Nepal [4].

Almost the entire cost of surgery is born by the hospital for the DST patients, including medicine, pre and post-operative care, and hospital stay. DST patients are only asked to pay for post-operative medicines (Nepali Rupees 100), dark glasses (Nepali Rupees 60) food during the hospital stay (Nepali Rupees 50), and transport home (Nepali Rupees 40) for a total of approximately US$3.

Comparing 2007/08 with 2006/07, the reduction in the number of DSTs by 50% had essentially no effect on the total number of cataract surgical procedures, presenting visual acuity or sex.

Bharatpur Eye Hospital was particularly interested in maintaining the cataract surgical sex ratio that favoured women. Individual studies [5-7] and a recent meta-analysis[8] have documented that cataract surgical utilization favours men and that this is the most significant contributor to the excessive burden of blindness born by women, world-wide [9]. Over the past five years comprehensive, gender specific community interventions directed by Bharatpur Eye Hospital, in affiliation with the Lumbini Eye Institute, have increased utilization of services by women and significantly reduced the burden of blindness born by women in these regions [10].

The number of people in the 50-59 year category increased from 384 to 629 people (64%) with approximately the same proportional increase for both men and women. The study offers no explanation for the increased number of younger people in the new DST model. In the context of the data from the following year, which showed a further increase to 965 people in this age group, it is quite plausible that the change is a historical trend related to increased awareness of the eye care programs in general and not related to the DST model changes in particular.

This study did not consider the presenting visual acuity by age group, however it seems reasonable to conclude that more younger people are a good trend as they are likely obtaining cataract surgery with less visual impairment. This is supported somewhat by data in Table 2 where 23% of people in year 2 had presenting visual acuity less than 6/18 versus 18% in year one. Operating on younger people with less visual impairment is not a problem for Bhartpur Eye Hospital as it has considerable capacity to increase its services. The risk, in other settings, is that younger people with less visual impairment could displace older people with blindness, particularly if the younger people pay for surgery directly.

The 22% decrease (236 people, from 1050 to 814) in utilization by men and women age 70 and older was of concern to Bhartpur Eye Hospital as people in this age group are typically among the most vulnerable populations in poorer countries and the population most likely to have severe visual impairment due to cataract. The decrease in utilization by people over age 70 could not be explained through available interview material or data on distance travelled. In fact, deficiencies in data led to counsellors and field staff focussing on data gathering in this age group in the subsequent year (2008-09). It seems reasonable to assume that decreased utilization reflects know barriers, including distance travelled and logistical problems arranging people to accompany them to fewer camps [3,5,6].

Before any changes could be implemented, in 2008-09 (data not shown) the number of people over age 70 who received cataract surgery rose to 992. Current estimates for 2009-10 are approximately 1100, numerically higher than the number (1050) operated in 2007. Although the decrease in annual utilization in this age group in 2007-08 is resolved, this does not mean that the current utilization by people in this age group is considered adequate. They remain one of the main target groups in which to continually increase utilization over time

This evaluation was limited to cataract services and did not consider all dimensions of providing primary eye care diagnosis and treatment. Other surgical procedures and other services such as treating minor injuries and providing corrective lenses are impacted by changes in DST patterns but were not considered herein.

## Conclusions

The new, more concentrated, more rural DST model of service delivery reduced overall outreach program costs, cost per cataract surgery transported, while increasing direct payments to the hospital, with a significant decrease in the number of people age 70 and older in the first year.

### Recommendations

1. Department specific (i.e. optical shop, dispensary) and program specific (i.e. cataract services) accounting systems are needed.

2. Improvement is needed in determining the individual procedure costs in hospital.

3. Data analysis should examine trends in age and visual acuity at presentation, particularly barriers to service utilization among people age 70 and older.

## Competing interests

The authors declare that they have no competing interests.

## Authors' contributions

RK and KB conceived of the study. SR and KB designed, RK, SR and MG carried out the study. SR and MG gathered and all authors reported data. All authors read and approved the final manuscript.

## Pre-publication history

The pre-publication history for this paper can be accessed here:

http://www.biomedcentral.com/1471-2415/10/9/prepub
